# Phosphomimetic Modulation of eNOS Improves Myocardial Reperfusion and Mimics Cardiac Postconditioning in Mice

**DOI:** 10.1371/journal.pone.0085946

**Published:** 2014-01-21

**Authors:** Terrence Pong, Marielle Scherrer-Crosbie, Dmitriy N. Atochin, Kenneth D. Bloch, Paul L. Huang

**Affiliations:** 1 Cardiovascular Research Center, Cardiology Division, Department of Medicine, Massachusetts General Hospital, Boston, Massachusetts, United States of America; 2 School of Engineering and Applied Sciences, Harvard University, Cambridge, Massachusetts, United States of America; 3 Harvard-MIT Division of Health Sciences & Technology, Cambridge, Massachusetts, United States of America; 4 Cardiac Ultrasound Laboratory, Cardiology Division, Department of Medicine, Massachusetts General Hospital, Boston, Massachusetts, United States of America; 5 Anesthesia Center for Critical Care Research, Department of Anesthesia, Critical Care, and Pain Medicine, Massachusetts General Hospital, Boston, Massachusetts, United States of America; University of Pittsburgh School of Medicine, United States of America

## Abstract

**Objective:**

Myocardial infarction resulting from ischemia-reperfusion injury can be reduced by cardiac postconditioning, in which blood flow is restored intermittently prior to full reperfusion. Although key molecular mechanisms and prosurvival pathways involved in postconditioning have been identified, a direct role for eNOS-derived NO in improving regional myocardial perfusion has not been shown. The objective of this study is to measure, with high temporal and spatial resolution, regional myocardial perfusion during ischemia-reperfusion and postconditioning, in order to determine the contribution of regional blood flow effects of NO to infarct size and protection.

**Methods and Results:**

We used myocardial contrast echocardiography to measure regional myocardial blood flow in mice over time. Reperfusion after myocardial ischemia-reperfusion injury is improved by postconditioning, as well as by phosphomimetic eNOS modulation. Knock-in mice expressing a phosphomimetic S1176D form of eNOS showed improved myocardial reperfusion and significantly reduced infarct size. eNOS knock-out mice failed to show cardioprotection from postconditioning. The size of the no-reflow zone following ischemia-reperfusion is substantially reduced by postconditioning and by the phosphomimetic eNOS mutation.

**Conclusions and Significance:**

Using myocardial contrast echocardiography, we show that temporal dynamics of regional myocardial perfusion restoration contribute to reduced infarct size after postconditioning. eNOS has direct effects on myocardial blood flow following ischemia-reperfusion, with reduction in the size of the no-reflow zone. These results have important implications for ongoing clinical trials on cardioprotection, because the degree of protective benefit may be significantly influenced by the regional hemodynamic effects of eNOS-derived NO.

## Introduction

Ischemic postconditioning is a modified form of reperfusion that results in reduced myocardial tissue damage [1], [2]. Postconditioning represents a particularly attractive therapy against myocardial ischemia/reperfusion (I/R) injury because it does not require foreknowledge of the ischemic event. The mechanisms underlying cardioprotection from postconditioning and preconditioning have been reviewed recently [3], [4], [5], [6], [7], and involve adenosine receptor signaling, and activation of pro-survival kinase pathways including ERK1/2, Akt/PI3 kinase, and STAT3. These pathways result in cellular changes, including activation of mitochondrial ATP-dependent K channels and inhibition of mitochondrial permeability transition pore opening, resulting in reduction in tissue damage. Beneficial effects from cardiac postconditioning have been shown in acute MI patients undergoing PCI [8], [9], [10], [11], [12]. Larger clinical trials are now underway for both postconditioning itself and for pharmacologic agents based on its underlying cardioprotective pathways [5], [7], [13], [14], [15]. eNOS and NO are involved in cardiac postconditioning signaling [16], [17], [18], [19], [20]. However, a direct role for eNOS in modulating regional myocardial blood flow during reperfusion has not been demonstrated.

Previous methods for measuring myocardial blood flow in mice do not have sufficient temporal or spatial resolution to follow regional myocardial perfusion following I/R injury. We used myocardial contrast echocardiography (MCE) of intravenously infused echogenic microbubbles to monitor blood flow in vivo in mice [21], [22]. Here, we use MCE to dynamically track regional myocardial blood flow during I/R to understand the mechanisms of postconditioning protection.

We use wild-type C57BL/6 mice, eNOS knockout mice, and eNOS mutant mice carrying a single amino acid mutation at the serine 1176 phosphorylation site (S1176D) [23]. Phosphorylation of eNOS at this serine (corresponding to serine 1177 in man) increases eNOS enzymatic activity and NO production [24], [25]. In S1176D mice, the codon for serine is replaced by one encoding aspartate to achieve a phosphomimetic gain-of-function mutation.

Our current results demonstrate that postconditioning is characterized by improved regional myocardial blood flow after I/R, and by an increase in eNOS S1176 phosphorylation. Further, the phosphomimetic eNOS mutation by itself improves blood flow and reduces infarct size. In contrast, eNOS knockout mice do not show reduction in infarct size or improvements in blood flow following postconditioning. These results reveal the importance of improved myocardial perfusion as a mechanism for protection from postconditioning. Further, they demonstrate that modulation of eNOS phosphorylation influences myocardial reperfusion, affecting tissue outcome from cardioprotection.

## Materials and Methods

### Animals

eNOS knockout mice and eNOS S1176D mutant mice were generated and genotyped as previously described [23], [26]. C57BL/6 mice (Jackson Laboratories) were used as wild-type controls. All animals were on C57BL/6 genetic background and 8–12 weeks at the time of the experiments.

### Ethics Statement

Experiments were approved by the Massachusetts General Hospital Institutional Animal Care and Use Committee (Permit 2003-N000297). All surgery was performed under sodium pentobarbital and ketamine anesthesia, and all efforts were made to minimize suffering.

### Nitrite/nitrate assay

eNOS enzymatic activity was assessed by measurement of nitrite and nitrate in myocardial tissue using a fluorometric assay (BioVision). Tissue samples were lysed for 10 minutes on ice with 300µl of tissue-lysis buffer. Tissue homogenates were centrifuged at 10,000 g for 5 minutes at 4°C and further filtered through a 10 kDa MW cut-off filter (BioVision). Filtrate was collected for the NO assay.

### In vivo myocardial ischemia reperfusion model

Animals were anesthetized with sodium pentobarbital (50 mg/kg ip) and ketamine hydrochloride (50 mg/kg ip). Depth of the anesthesia was moni­tored by tail pinch, respiratory rate and heart rate. Mice were intubated and ventilated. Left anterior descending coronary artery (LAD) ligation was performed through a thoracotomy. A 7-0 silk ligature was passed under­neath the vessel and through a custom snare to induce ischemia without damaging the artery. Ischemia was achieved by tightening the snare for 45 minutes and occlusion was confirmed by observed blanching of the anterior left ventricular wall. After 45 minutes, the myocardium was reperfused with one of two algorithms 1) traditional myocardial ischemia-reperfusion (MIR) injury where the ligation was simply released or, 2) myocardial ischemia reperfusion with postconditioning (MIPc) where six cycles of 10 sec reperfusion was followed by 10 sec of ischemia. Buprenorphine HCl (0.05–0.1 mg/kg) was administered post-operatively.

### Infarct size determination

After 24 hour reperfusion, the LAD was religated with 7-0 silk suture, and 1 ml of 1% Evans blue was perfused retrograde through the left carotid artery to delineate the area at risk (AAR). The heart was excised and fixed in a 2% solution of agarose gel and allowed to solidify. Myocardial tissue was sectioned into 1-mm-thick axial sections. Infarct size was determined by staining with 2,3,5-triphenyltetrazolium chloride (TTC) for 20 minutes in the dark at 37°C. Each sliced was weighed and photographed through a dissecting microscope. The left ventricular area, AAR, and area of infarction for each slice were determined by planimetry. The final size of infarction was determined by integrating the infarct areas in each myocardial slice over the entire myocardium as previously described [27].

### Western blot analysis

Myocardial tissues were harvested after 45 min ischemia and 10 min reperfusion. The left ventricular AAR was identified by blanched myocardium during ischemia. Myocardial tissues were homogenized to obtain protein extracts and 100µg of protein was subjected to electrophoresis in 7% Tris-HCl polyacrylamide gels. Proteins were transferred to PDVF membranes for Western blot analysis and visualized by chemiluminescence. Antibodies to eNOS (Sigma-Aldrich), phospho-eNOS (p-Ser1177 human sequence numbering, BD Bioscience), Akt (Cell Signaling) and phospho-Akt (p-Ser473, Cell Signaling) were obtained commercially.

### Myocardial contrast echocardiography

MCE studies were performed as previously described [22]. Mice were anesthetized with intraperitoneal injections of sodium pentobarbital (50 mg/kg) and ketamine hydrochloride (50 mg/kg). Heart rate and blood pressure were monitored through a carotid catheter and recorded with a blood pressure analysis module in PowerLab (ADInstruments). A venous line was placed in the left jugular vein for constant infusion of microbubbles. 10% Perflutren lipid microspheres (Definity, Lantheus Imaging) were diluted tenfold in sterile saline and infused intravenously at 20µL/min. A thoracotomy was performed to provide unobstructed visualization of the myocardium. A 7-0 silk suture was passed underneath the LAD and through a custom snare. MCE was performed with a 14 MHz linear transducer using an Acuson Sequoia C512 system, using a mechanical index of 0.24. Perfusion images were obtained in real time following destruction of microbubbles using a sequence of 10 high-energy frames (mechanical index 1.9). Signal intensity was obtained for 10 seconds after the high-energy sequence at a frame rate of 30 Hz. Parasternal long-axis views were recorded at the level of the aortic arch. For temporal monitoring of myocardial blood flow MCE was used to measure blood flow at baseline before ischemia, during ischemia, and at 5, 10, 30 minutes after initiation of reperfusion.

### Myocardial perfusion analysis

MCE was performed on the anteroseptal wall in the parasternal long-axis view using methods previously described [22]. Detection and segmentation of the left ventricular septum was accomplished using a shape based snake model of edge detection in Matlab (Mathworks) [28], [29]. The anterior septum was divided into three regions of interest defined by the apical septum, mid septum and basal septum. Average signal intensity within each region of interest was measured in each frame and a curve of signal intensity over time was fitted to an exponential function: *y  =  A (1* – *e^−β t^),* where y is the signal intensity, β is the initial slope of the curve and A is the signal plateau intensity. Two to three curves were averaged per time-point for each animal. Myocardial blood flow was estimated by the product of Aβ. Values are expressed as percentage of baseline Aβ.

### No-reflow analysis

MCE measurements were taken at baseline as a measure of myocardial blood flow before ischemia and compared against MCE measurements taken at the end of the study, 30 minutes after reperfusion. Three consecutive MCE images were averaged for each animal under each condition, the average signal intensity of the left ventricular anteroseptum at baseline was calculated. Images were converted to relative myocardial blood flow images by using a thresholding paradigm to identify areas of severe (0%–20% residual blood flow, representing the core ischemic region) and moderate (21%–30% residual blood flow, representing penumbra) blood flow relative to baseline [30].

### Statistics

All results are expressed as mean ± SD, except western blot densities which are expressed as mean ± SEM. Statistical analysis was performed in Matlab (Mathworks) using Kriskal-Wallis analysis of variance and Wilcoxon rank-sum tests with Bonferonni’s correction. Differences of P<0.05 were considered significant.

## Results

### Postconditioning and S1179D mice show protection from I/R injury in vivo

**Figure 1:**
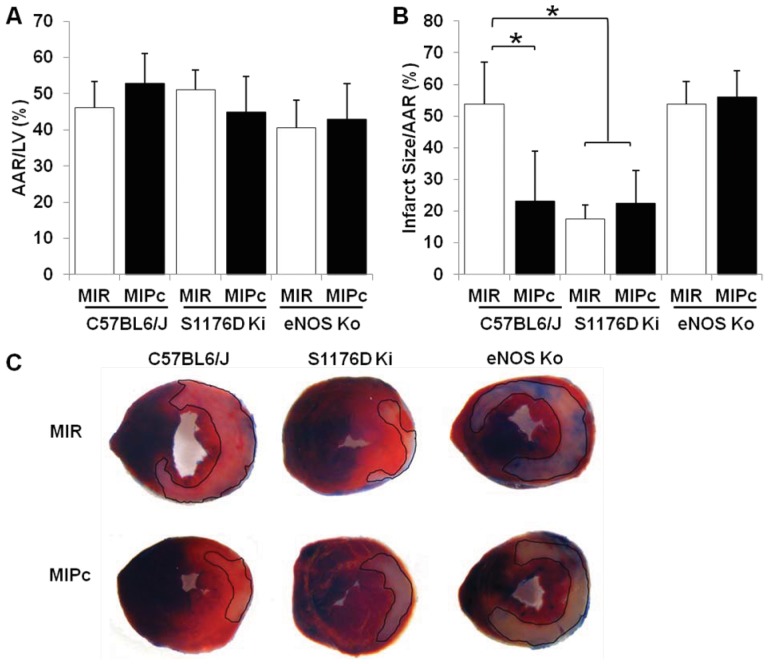
eNOS S1176 phosphorylation protects against I/R injury in vivo. Wild-type, S1176D and eNOS knockout mice were subjected to 45 minutes of myocardial ischemia (LAD ligation) followed by traditional reperfusion (MIR) or postconditioned reperfusion (MIPc: 6 cycles of 10sec reperfusion, 10 sec ischemia). **A.** Percentage of left ventricle area at risk (AAR), (P = NS). **B.** Quantitative analysis of infarct size over AAR, *P<0.05 compared to wild-type control. **C.** Representative heart sections perfused with 1% Evans blue and stained with 2% TTC; infarct areas are outlined in black. MIR: Myocardial ischemia with reperfusion, MIPc: Myocardial ischemia with postconditioning, AAR: Area at Risk, LV: Left Ventricle. n = 6–9 mice per group. Data are expressed as the mean±SD.

We examined the response to myocardial I/R injury in wild-type mice, S1176D mice, and eNOS knockout mice by ligation of the left anterior descending (LAD) artery for 45 minutes, followed by reopening of the ligated vessel. We compared the response of animals subjected to myocardial ischemia reperfusion alone (MIR) with those subjected to myocardial ischemia reperfusion with postconditioning (MIPc), using a pattern of 6 cycles of 10 sec reperfusion followed by 10 sec ischemia. The areas at risk, determined 24 hours after I/R, were not significantly different across groups (Figure 1A). WT mice subjected to MIPc developed smaller infarcts (ratio of infarct size/area at risk) compared to mice subjected to MIR (Figure 1B left). S1176D mice displayed smaller infarcts in both MIR and MIPc groups (Figure 1B middle). The reduced infarct sizes in S1176D ki mice were comparable to those seen in postconditioned WT mice. eNOS ko mice showed no reduction in infarct size from postconditioning (Figure 1B right). Representative infarct slices with the infarct zones outlined are shown in Figure 1C.

### eNOS protein levels and enzymatic activity in mutant mice

**Figure 2:**
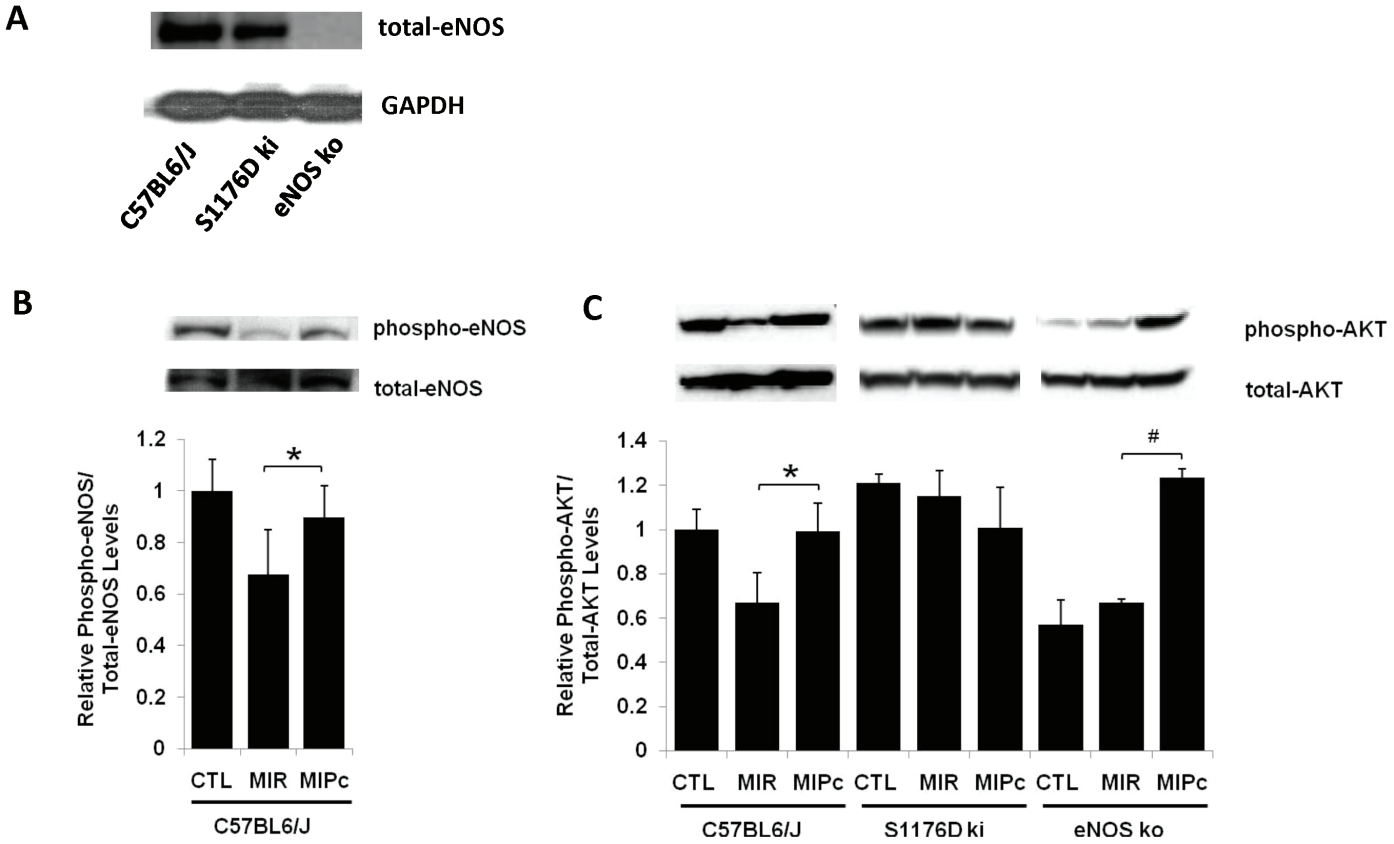
Postconditioning activates Akt and eNOS. **A.** Western blot of total eNOS and GADPH in WT (C57BL6/J), S1176D mice (S1176ki), and eNOS ko mice. **B.** Western blot demonstrating phosphorylation (Ser1176) and total protein levels of eNOS in wild-type mice under conditions of control (CTL), MIR, and MIPc. **C.** Representative Western blot demonstrating phosphorylated (Ser473) and total protein levels of Akt. Densities (arbitrary units, AU) show that MIPc phosphorylates Akt in WT and eNOS ko mice. n = 5 per group. Data are expressed as the mean±SD. *P<0.05.

Total eNOS protein levels were the same in the hearts of WT and S1176D mice, and eNOS was not detectable in the hearts of eNOS ko mice (Figure 2A). However, eNOS enzymatic activity, as reflected by fluorometric determination of nitrate/nitrite stable breakdown products of NO metabolism, was greater in heart tissue of S1176D mice than of WT mice (3.2 pmol/mg tissue vs. 2.5+/–0.6 pmol/mg tissue, p = 0.03), consistent with results from other tissues [23]. Thus, baseline levels of eNOS enzymatic activity and NO in the myocardium can be regulated by phosphomimetic modulation of the S1176 phosphorylation site.

### Postconditioning activates Akt and eNOS

We determined the expression levels and degree of phosphorylation of Akt and eNOS following in response to myocardial I/R injury. Figure 2B shows the effects of MIR and MIPc on eNOS phosphorylation, all in WT mice. MIR diminishes eNOS phosphorylation at S1176, while MIPc does not. Figure 2C shows the effects of I/R and postconditioning on Akt phosphorylation. Akt phosphorylation at S473 was higher in wild-type mice after MIPc than MIR alone. In wild-type mice, these changes are associated with parallel changes in eNOS phosphorylation at S1176 (Figure 2B). In S1176D mice, the level of Akt phosphorylation did not change with MIR or MIPc (Figure 2C, middle). In eNOS knockout mice, Akt phosphorylation was increased following MIPc (Figure 2C, right), though this increase, in the absence of eNOS, did not result in cardioprotection (Figure 1B, right). The reasons for increased Akt phosphorylation in eNOS ko mice is not known but possibilities include positive feedback between eNOS phosphorylation and phosphorylation of Akt kinase itself.

### Postconditioning eNOS and S1176 phosphorylation are associated with improved regional myocardial perfusion kinetics

**Figure 3:**
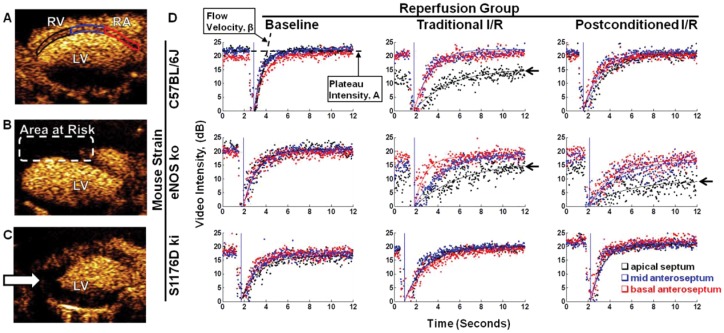
MCE of regional blood flow following I/R. **A.** The left ventricular septum in the parasternal long axis view was divided into three regions of interest: apical septum (black), mid septum (blue) and basal septum (red). RV: Right ventricle, RA: Right atrium, LV: Left ventricle. Representative MCE image taken at baseline, **B.** Representative MCE image during ischemia. **C.** Representative MCE image 30 minutes after reperfusion. **D.** Analysis of regional change in myocardial perfusion. Representative region-specific replenishment curves 30 minutes post-reperfusion are shown: apical septum (black), mid septum (blue), and basal septal regions (red). Replenishment curves are characterized by the myocardial blood flow parameters A (plateau intensity) and β (flow velocity).

We used MCE to dynamically track regional myocardial blood flow [21], [22] during MIR and MIPc. Wild-type, eNOS knockout, and S1176D mice were subjected to 45 min LAD ligation and reperfusion, without (MIR) and with postconditioning (MIPc) to determine whether improvements in regional myocardial blood flow could contribute to cardioprotection. We obtained high quality two-dimensional parasternal long-axis images of the myocardium to follow regional myocardial perfusion (Figure 3A). The anterior septum was divided into three regions to capture the distinct blood flow changes in each anatomic region. Region 1,outlined in red, is the basal anteroseptum. Region 2, outlined in blue, is the mid anteroseptum. Region 3, outlined in black, is the apical septum. The site of LAD ligation is located between regions 2 and 3. The majority of blood flow deficits are found in the apical septum.

After contrast containing microbubbles are introduced into the circulation, the bubbles are synchronously destroyed in the field of view, and their reappearance is quantitated over time in specific regions. Two parameters describe regional perfusion. The first parameter is A, the steady state level (plateau) of the microbubbles (see Figure 3D). The second parameter is β, that rate of reappearance (slope) of microbubbles following synchronous destruction [31]. Regional myocardial blood flow was estimated by the product of these two parameters (Aβ) [22], [32].

In wild-type mice (Figure 3D, top row), baseline blood flow (left panel) in all three regions is comparable. With MIR (middle panel), perfusion was markedly impaired in the apical septum (black, arrow) in the region corresponding to the eventual infarct. With MIPc, (right panel), perfusion in the apical septum (black) is improved over with MIR. These results show that MIPc is associated with improvements in the restoration of regional blood flow in the ischemic zone.

In eNOS knockout mice (Figure 3D, middle row), baseline blood flow (left panel) in all three regions is comparable, and similar to those seen in wild-type mice. With MIR, regional blood flow is reduced in both the mid-septum (blue) and apical septum (black). With MIPc, the blood flow in these regions continues to be poor, and is even worse than with MIR. These results show that eNOS knockout mice have more pronounced regional perfusion defects than wild-type mice, and unlike wild-type mice, they show no improvements in regional blood flow with postconditioning.

In contrast, S1176D mice (Figure 3D, bottom row) showed robust tolerance against myocardial blood flow deficit caused by MIR. Replenishment curves from both MIR (middle panel) and MIPc (right panel) groups showed minimal deficits in myocardial blood flow. These results indicate that the phosphomimetic eNOS mutation by itself is associated with improved restoration of regional perfusion.

**Figure 4:**
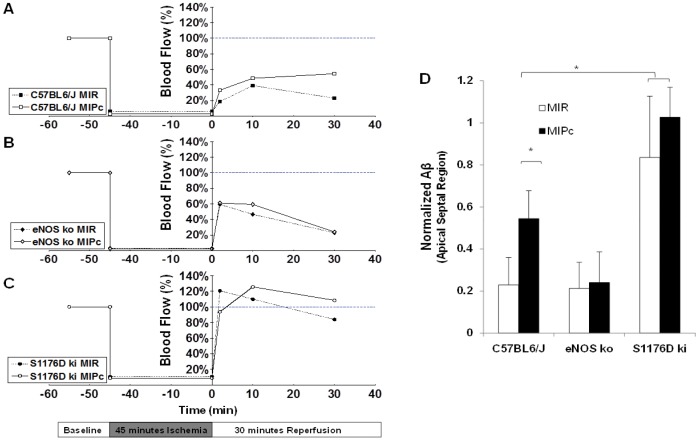
Temporal myocardial contrast echocardiography. Myocardial blood flow (Aβ) profiles in the apical region for **A.** C57BL/6J mice. **B.** eNOS knockout mice, and **C.** S1176D knockin mice. Levels of myocardial myocardial blood flow were normalized to baseline values and measured at 2, 10 and 30 minutes after reperfusion. **D.** Apical myocardial blood flow 30 minutes after reperfusion. MIR: Traditional myocardial ischemia with reperfusion, MIPc: Myocardial ischemia with postconditioning. n = 5–6 per group. Data are expressed as the mean±SD. *P<0.05.

To extend our understanding of the temporal dynamics of the apical blood flow deficits associated with MIR and MIPc, we performed MCE serially over the time-course of reperfusion. Figure 4 shows temporal MCE profiles in the apical septum for wild-type, eNOS knockout, and S1176D mice. Blood flow measurements were normalized to myocardial blood flow values at baseline. Wild-type mice subjected to MIR exhibited worsening myocardial blood flow over time, whereas WT mice subjected to MIPc showed improvement in myocardial blood flow, with significantly improved perfusion after 30 minutes (Figure 4A). eNOS knockout mice showed worsening myocardial blood flow over time with both MIR and MIPc, with no detectable difference at 30 minutes (Figure 4B). Although the 1 minute timepoint shows a higher blood flow in the S1176D mice (Figure 4B) than in the WT mice (Figure 4A0, this early difference did not translate to improvement in blood flow at 30 minutes, nor was there a difference between MIR and MIPc groups. S1176D mice show rapid return to pre-ischemic levels of perfusion and higher with both MIR and MIPc (Figure 4C). Quantitation of the myocardial blood flow in the apical septum at 30 minutes of reperfusion (Figure 4D) shows that MIPc is associated with improvement in reperfusion in wild-type mice. eNOS knockout mice fail to show reperfusion with either MIR or MIPc. S1176D mice show robust reperfusion with both MIR and MIPc. The temporal results from MCE analysis support the notion that increased phosphomimetic eNOS activity enhances myocardial blood flow after I/R injury. Mean arterial pressure and heart rate were monitored throughout the course of I/R and no significant difference was found between groups (data not shown).

### Assessment of myocardial no-reflow zones

**Figure 5:**
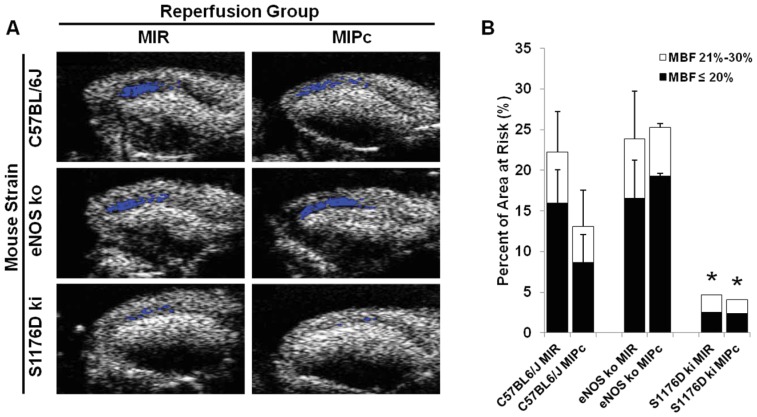
Effect of postconditioning and S1176D mutation on no-reflow zones. **A.** Representative images 30 minutes after reperfusion. Superimposed areas (blue) indicate regions with ≤20% residual blood flow. **B.** Composite graph showing areas of the myocardium with ≤20% (black) and ≤30% (white) residual blood flow compared to preischemic baseline. MBF: myocardial blood flow. n = 5–6 per group. Data are expressed as the mean±SD. *P<0.05.

Despite opening of an infarct-related artery, no-reflow zones may result from persistent perfusion defects. These are thought to be caused by obstructed or dysfunctional capillaries in the microvasculature caused by endothelial dysfunction. We assessed the spatial extent of no-reflow zones using MCE, defined as regions with severe blood flow reductions to less than 20% of baseline blood flow [33]. As shown in Figure 5, wild-type mice developed larger no-reflow zones at the 30 minute timepoint after MIR than after MIPc. eNOS knockout mice showed large no-reflow zones comparable to wild-type mice treated with MIR. S1176D mice showed significantly smaller no-reflow zones with both MIR and MIPc.

## Discussion

Much of the clinical treatment of acute myocardial infarction focuses on restoring coronary artery blood flow through the infarct vessel, either through pharmacologic agents (thrombolysis, antiplatelet agents) or mechanical means (percutaneous coronary intervention or coronary artery bypass graft surgery). However, reperfusion following ischemia can be associated with significant tissue damage, due to rapid normalization of pH, Ca^2+^ overload, generation of reactive oxygen species, and opening of mitochondrial permeability transition pores [6]. Reperfusion injury can be reduced by cardiac preconditioning and postconditioning paradigms. Postconditioning is particularly attractive because it does not require foreknowledge of the ischemic event. The mechanisms of preconditioning and postconditioning involve the activation of cardioprotective survival pathways, including ERK1/2 and Akt kinase (together termed the reperfusion injury salvage kinases or RISK pathways) and TNFα and STAT3 pathways (together termed the survival activating factor enhancement or SAFE pathways). These pathways lead to activation of the mitochondrial ATP-dependent potassium channel and inhibition of mitochondrial permeability transition pore opening, resulting in decreased cell death [5], [6], [7], [13], [34]. eNOS is known to be activated by these cardioprotective pathways, and is thought to play signaling roles in mediating postconditioning protection [17], [18], [19]. Despite its well known role as an endogenous vasodilator, a direct role for eNOS-derived NO in improving microvascular regional blood flow after postconditioning has not been demonstrated.

A unique aspect of the current report is the application of MCE to delineate regional myocardial blood flow over time during I/R and postconditioning. Our results show that despite opening of the infarct artery during reperfusion, MIR results in detectable defects in microvascular tissue perfusion in the distal septum following LAD ligation. These defects are less marked following MIPc, with improved tissue perfusion in the affected area. This establishes that in addition to cardioprotective signaling mechanisms, differences in restoration of regional blood flow contribute to the reduction in infarct size following postconditioning. Furthermore, Western blot results confirm that MIPc is associated with activation of Akt kinase and eNOS phosphorylation in wild-type mice. Interestingly, Akt phosphorylation is still observed in eNOS knockout mice, although the protective effects of postconditioning are lost in the absence of eNOS.

eNOS activity is regulated in vivo by a variety of mechanisms [35], [36], including phosphorylation at S1176, resulting in increased enzymatic activity [24], [25]. eNOS phosphorylation is deficient in diabetes and hyperlipidemia, and may mechanistically contribute to endothelial dysfunction seen in these conditions. Here, we use eNOS mutant mice that carry the S1176D gain of function mutation [23]. We previously showed that this phosphomimetic mutation rescues impaired blood flow in Akt1 deficient mice subjected to wound healing assays [37], and improves vessel reactivity and decreases stroke size when challenged with cerebral ischemia [38].

eNOS S1176D mice, even without postconditioning, show tolerance against I/R injury *in vivo*, comparable to postconditioned wild-type mice. MCE replenishment curves confirm that restoration of myocardial reperfusion in the area at risk is significantly improved in S1176D mice as compared with wild-type mice, both with MIR and MIPc. In contrast, eNOS knockout mice do not show any improvement from postconditioning, and MCE replenishment curves show more pronounced defects in reperfusion, not only in the apical septum, but also in the mid-septum when compared with wild-type mice.

The no-reflow zone is reduced by postconditioning in wild-type mice, but not in eNOS knockout mice. The no-reflow zone is markedly reduced in the S1176D mice, both with and without postconditioning. The finding that Akt phosphorylation is still increased in eNOS knockout mice by MIPc, while postconditioning protection is not observed, suggests that eNOS activity is required for the protective effects of Akt pathway activation. Further, these results suggest that effects on regional microvascular blood flow and reperfusion, mediated by eNOS, may interact with known cardioprotective mechanisms to modulate tissue outcome, and that cardioprotection may require a minimum degree of reperfusion to salvage tissue.

Our results here in eNOS ko mice contrast with a previous study that showed that eNOS ko mice develop larger infarcts than do WT mice after cardiac ischemia [27]. These differences are likely due to the specific experimental protocols used. The MIR model used here is a severe model of ischemia caused by LAD ligation for 45 minutes followed by reperfusion for 24 hours. Infarct size is over 50% of the ischemic zone for both WT and eNOS ko mice. In contrast, the previous study used a less severe model, with ischemia for 20 minutes followed by reperfusion for 120 minutes. Infarct size was 20.9% of the ischemic zone for WT mice, while it was 46% for eNOS ko mice [27].

In summary, our results establish that postconditioning improves restoration of myocardial blood flow in the area at risk, and increases eNOS S1176 phosphorylation in wild-type mice. These results are important because they demonstrate the *in vivo* effects of eNOS activity on microvascular blood flow during reperfusion. In addition to roles for NO in signaling and activation of pro-survival pathways, our findings reveal that eNOS influences the degree of myocardial reperfusion following I/R injury. These new insights suggest that eNOS S1176 activation and regional reperfusion dynamics could affect the degree of myocardial survival, relevant not only to postconditioning, but also to pharmacologic modulation of cardioprotective mechanisms now in clinical trials.
